# A humanized orthotopic mouse model for preclinical evaluation of immunotherapy in Ewing sarcoma

**DOI:** 10.3389/fimmu.2023.1277987

**Published:** 2023-10-06

**Authors:** Wen Luo, Hai Hoang, Yanling Liao, Jian Pan, Janet Ayello, Mitchell S. Cairo

**Affiliations:** ^1^ Department of Pediatrics, New York Medical College, Valhalla, NY, United States; ^2^ Department of Pathology, Immunology and Microbiology, New York Medical College, Valhalla, NY, United States; ^3^ Department of Medicine, New York Medical College, Valhalla, NY, United States; ^4^ Department of Cell Biology and Anatomy, New York Medical College, Valhalla, NY, United States

**Keywords:** humanized mouse model, orthotopic model, cord blood, immunotherapy, bone sarcoma, pediatric cancer

## Abstract

The advent of novel cancer immunotherapy approaches is revolutionizing the treatment for cancer. Current small animal models for most cancers are syngeneic or genetically engineered mouse models or xenograft models based on immunodeficient mouse strains. These models have been limited in evaluating immunotherapy regimens due to the lack of functional human immune system. Development of animal models for bone cancer faces another challenge in the accessibility of tumor engraftment sites. Here, we describe a protocol to develop an orthotopic humanized mouse model for a bone and soft tissue sarcoma, Ewing sarcoma, by transplanting fresh human cord blood CD34^+^ hematopoietic stem cells into young NSG-SGM3 mice combined with subsequent Ewing sarcoma patient derived cell engraftment in the tibia of the humanized mice. We demonstrated early and robust reconstitution of human CD45^+^ leukocytes including T cells, B cells, natural killer cells and monocytes. Ewing sarcoma xenograft tumors successfully orthotopically engrafted in the humanized mice with minimal invasive procedures. We validated the translational utility of this orthotopic humanized model by evaluating the safety and efficacy of an immunotherapy antibody, magrolimab. Treatment with magrolimab induces CD47 blockade resulting in significantly decreased primary tumor growth, decreased lung metastasis and prolonged animal survival in the established humanized model. Furthermore, the humanized model recapitulated the dose dependent toxicity associated with the CD47 blockade as observed in patients in clinical trials. In conclusion, this orthotopic humanized mouse model of Ewing sarcoma represents an improved platform for evaluating immunotherapy in bone and soft tissue sarcoma, such as Ewing sarcoma. With careful design and optimization, this model is generalizable for other bone malignancies.

## 1 Introduction

Preclinical small animal models are widely utilized for cancer research especially for evaluation of the safety and efficacy of anti-tumor regimens prior to clinical trials in human patients for low cost and ethical reasons. These small animal models, mostly syngeneic or genetically engineered mouse models, are capable of mimicking many human cancers, because these mice follow similar disease progression as human tumors, making them invaluable tools for cancer research, albeit their inherent limitations largely secondary to the genetic, physiologic and immunologic differences compared to human.

Ewing sarcoma (ES) is a highly metastatic bone and soft tissue tumor that mostly occurs in children, adolescents and young adults. Despite multiple therapeutic approaches including surgery, radiation and chemotherapy, the outcome of patients with metastatic ES has remained dismal (less than 25% overall survival) over the past 40 years (1–3). A challenge for ES research is the lack of ideal small animal models. ES only arises in humans which excludes the possibility of developing syngeneic animal models. Multiple recent attempts from different groups to develop a EWS-FLI1-driven transgenic mouse model of ES have also failed (4). Current animal models for ES are mostly subcutaneously injected localized xenograft tumors in immunodeficient mouse hosts (nude, NOD-SCID or NSG). These models fail to reflect the highly metastatic nature of this bone and soft tissue tumor, because ES cells injected subcutaneously do not metastasize. Nor do these models recapitulate the tumor-immune cell interactions or tumor microenvironment in human patients due to the lack of human immune system in these mice. With the recent development of immunotherapy as a promising treatment modality for ES (5, 6), a new improved orthotopic mouse model capable of better evaluation of immunotherapy in ES is urgently needed and represents an unmet need.

Recently, humanized mouse models have been developed for a variety of human cancers, including breast, colorectal, pancreatic, lung, adrenocortical, melanoma and hematological malignancies (7–12). These humanized mice harbor human hematopoietic and immune cells and are considered a feasible and ideal model to study cancer immunology and evaluate cancer immunotherapy. However, few orthotopic humanized mouse models are currently reported for bone cancers (13) such as ES (14). Furthermore, these humanized models are not ideal to study pediatric cancers because the mice already reach adulthood when the reconstitution of human immune system occurs.

In the present study, using cord blood derived CD34^+^ human hematopoietic stem cells (HSC), we established human immune system in young NSG-SGM3 mice. We successfully engrafted ES tumors in humanized NSG-SGM3 mice by orthotopic intratibial transplantation of patient derived ES cells. We further validated the utility of this model by comparing the efficacy of magrolimab, a potentially effective immunotherapy agent in ES, in the humanized orthotopic model and the non-humanized counterpart. With careful design and optimization, our approach can be generalized to other human bone cancers.

## 2 Materials and reagents

### 2.1 Reagents

- NSG-SGM3 (NOD.Cg-Prkdcscid Il2rgtm1Wjl Tg(CMV-IL3,CSF2,KITLG)1Eav/MloySzJ) mice, males or females, 3 to 4 weeks old (013062, The Jackson Laboratories, Farmington, CT), housed in microisolator (filter bonneted) or pressurized, individually ventilated cages.
- Cord blood (Vitalant, Pittsburgh, PA).
- Ficoll-Paque ^™^ PLUS Media (45-001-750, Fisher Scientific, Waltham, MA).
- Human CD34 MicroBead Kit (130-046-702, Miltenyi Biotec, Gaithersburg, MD).
- 1-cc tuberculin syringes (14-826-87, Fisher Scientific, Waltham, MA).
- 30 Gauge hypodermic needles (14-821-12A, Fisher Scientific, Waltham, MA).
- BD microtainer blood collector (lavender) (02-669-33, Fisher Scientific, Waltham, MA).
- Microhematocrit capillary tubes (22-362566, Fisher Scientific, Waltham, MA).
- Sterile petrolatum ophthalmic ointment (NC2004680, Fisher Scientific, Waltham, MA).
- Antibodies for flow cytometry and immunofluorescent analyses (see **Table 1**).
- FACS buffer: 1xDPBS (Fisher Scientific, 14-190-250), 0.5% BSA (130-091-376, Miltenyi Biotec, Gaithersburg, MD).
- Red blood cell lysis buffer (420301, BioLegend, San Diego, CA).
- 12 x 75-mm round bottom tubes for flow cytometry.
- Magrolimab (Gilead Sciences Inc., San Dimas, CA).
- A673 ES cell line (CRL-1598, American Type Culture Collection, Manassas, VA).
- Mammalian expression construct for Luciferase (pMMP-LucNeo, Stephen Lessnick, MD, Nationwide Children’s Hospital, Columbus, OH).
- Dulbecco’s Modified Eagle’s Medium (DMEM, MT10013CV, Fisher Scientific, Waltham, MA).
- Roswell Park Memorial Institute Medium (RPMI, 10-040-CV, Corning, Corning, NY).
- Fetal Bovine Serum (FBS, 16000044, Thermo Fisher Scientific, Waltham, MA).
- Penicillin-Streptomycin-Glutamine (10378016, Thermo Fisher Scientific, Waltham, MA).
- Trypsin-EDTA (0.25%) (25-200-114, Fisher Scientific, Waltham, MA).
- Dulbecco’s phosphate-buffered saline (DPBS, 14190250, Thermo Fisher Scientific, Waltham, MA).
- Geneticin™ Selective Antibiotic (Neomycin) (10131035, Thermo Fisher Scientific, Waltham, MA).
- Matrigel^®^ Growth Factor Reduced (GFR) Basement Membrane Matrix, LDEV-free (354230, Corning, Corning, NY).
- Hamilton™ CTC and LEAP Technologies GC PAL Autosampler Syringes (14-685-520, Fisher Scientific, Waltham, MA).
- Povidone-Iodine swab (Novaplus V9123 10% solution, NC0436628, Fisher Scientific, Waltham, MA).
- D-Luciferin, Potassium Salt (LUCK-1G, Gold Biotechnology, Olivette, MO).
- Tissue-Tek O.C.T. Compound (4583, Sakura Finetek USA, Torrance, CA).
- Glass Microscope Slides (1358W, Globe Scientific INC, Mahwah, NJ).
- Micro cover glasses (48404-453, VWR, Radnor, PA).
- Paraformaldehyde (PFA, 158127, Millipore Sigma, Burlington, MA).
- VECTASHIELD antifade mounting medium (H-1200-10, Vector laboratories, Costa Mesa, CA).

**Table 1:** Antibodies for flow cytometry and immunofluorescent analyses.

Antigen	Fluorescein	Clone	Catalog #	Isotype control	Catalog #	Manufacturer
Mouse CD45	FITC	30-F11	553079	rat IgG2b, κ	553988	BD Biosciences
	PE	30-F11	553081	rat IgG2b, κ	553989	BD Biosciences
	PerCP	30-F11	557235	rat IgG2b, κ	552991	BD Biosciences
	APC	30-F11	559864	rat IgG2b, κ	553991	BD Biosciences
	BV421	30-F11	103133	rat IgG2b, κ	400639	BioLegend
Human CD45	APC	HI30	555485	mouse IgG1, k	555751	BD Biosciences
Human CD3	PE	UCHT1	555333	mouse IgG1, k	555749	BD Biosciences
Human CD20	BV421	2H7	562873	mouse IgG2b, κ	562748	BD Biosciences
Human CD56	FITC	HCD56	318304	mouse IgG1, k	400109	BioLegend
Human CD34	FITC	581	343503	mouse IgG1, k	400107	BioLegend
Mouse F4/80			123101			BioLegend
Human CD68			14-0688-82			ThermoFisher
Human CD47			14-0479-82			ThermoFisher

### 2.2 Equipment

- RS 2000 small animal Irradiator (Rad Source Technologies) or any ^137^Cs gamma irradiator with autoclaved, filtered and ventilated device in irradiator chamber for housing mice during irradiation.
- Class II biological safety cabinet.
- BD FACSCelesta Cell Analyzer (BD Biosciences, La Jolla, CA) or any flow cytometer capable of five-color cytometry.
- IVIS Spectrum *In Vivo* Imaging System (124262, PerkinElmer, Waltham, MA).
- Isoflurane vaporizer for small animals (Ohmeda Isotec 4).
- CM1850 cryostat (Leica Biosystems, Deer Park, IL).
- EVOS M5000 imaging system (Thermo Fisher Scientific, Waltham, MA) or any fluorescent microscope.

## 3 Methods

### 3.1 Humanization of NSG-SGM3 mice with CD34^+^ HSC from human cord blood

All animal studies need to be performed in accordance with protocols approved by the Institutional Animal Care and Use Committee.

Place 3–4-week-old NSG-SGM3 mice into a sterile, filtered, ventilated device for housing during irradiation. Irradiate mice with 100 cGy sublethal whole body gamma irradiation. Return the mice to their microisolator cages, provide proper food and water and wait for 24 hours before injecting the CD34^+^ HSCs.

Isolate mononuclear cells from cord blood by ficoll-paque density gradient centrifugation. Isolate CD34^+^ HSCs from Ficoll-isolated cells using Human CD34 MicroBead Kit according to the manufacturer’s instructions. Resuspend CD34^+^ HSCs in DPBS at 2 x 10^6^ cells/mL and immediately inject 100 μL containing 2 x 10^5^ CD34^+^ HSCs into the lateral tail vein of irradiated mice using a 1-cc tuberculin syringe with 30 Gauge needles. Frozen-thawed CD34^+^ HSCs may also be used with the price of lower chimerism than fresh cells. Allow HSCs to engraft for a minimum of 4 weeks. Draw blood from the mice and perform flow cytometry analysis to evaluate engraftment levels 4, 6, and 8 weeks after HSC injection.

### 3.2 Flow cytometry analysis of humanized mice

Titrate all anti-human antibodies (**Table 1**) on a 1:5 mixture of human peripheral blood mononuclear cells (PBMC) and splenocytes from non-engrafted NSG-SGM3 mice to ensure specificity and no cross-reactivity with mouse cells.

Collect at least 150 μL whole blood via venous sinus, facial vein or tail vein from all mice to be tested, including a whole blood sample from a non-engrafted NSG-SGM3 mouse as a negative control.

At the end of the humanization evaluation, isolate splenocytes from the spleen and immune cells from bone marrow of all mice. Briefly, to collect splenocytes, using a 1 mL syringe plunger, gently press the spleen tissue pieces through a 70 μm cell strainer fitted on a 50 mL tube, while continuously adding RPMI media with 10% FBS. To harvest bone marrow, cut the ends of the femur off, using a syringe containing 5 mL RPMI media with 10% FBS, carefully flush out the marrow into a collection tube. Spin to collect the cells and resuspend in 200 μL DPBS.

Aliquot 100 μL of each sample (blood, splenocyte or bone marrow) into separate 12 x 75 mm tubes as “experimental” samples (**Table 2**).

**Table 2:** Flow cytometry antibody panel for evaluation of human engraftment in NSG-SGM3 mice.

Sample #	Sample	FITC	PE	PerCP	APC	BV421
1	Pooled	—	—	—	—	—
2	Pooled	mCD45	—	—	—	—
3	Pooled	—	mCD45	—	—	—
4	Pooled	—	—	mCD45	—	—
5	Pooled	—	—	—	mCD45	—
6	Pooled	—	—	—	—	mCD45
7	Pooled	mIgG2b, k	hCD3	mCD45	hCD45	hCD20
8	Pooled	hCD56	mIgG1, k	mCD45	hCD45	hCD20
9	Pooled	hCD56	hCD3	rIgG2b, κ	hCD45	hCD20
10	Pooled	hCD56	hCD3	mCD45	mIgG2b, k	hCD20
11	Pooled	hCD56	hCD3	mCD45	hCD45	mIgG2b, k
11+	Experimental	hCD56	hCD3	mCD45	hCD45	hCD20

Combine the remaining samples into a “pooled” sample and aliquot 50 μL of the “pooled” sample into separate 12 x 75 mm tubes for single color and unstained compensation controls as well as fluorescence minus one (FMO) controls (**Table 2**).

Stain with antibodies in the dark at room temperature for 1/2 hour (**Table 2**). Lyse red blood cells using lysis buffer according to the manufacturer’s instructions. Fix samples with 2% PFA and proceed to flow cytometry analysis or store at 4°C in the dark for up to 48 hours.

Collect data on all viable cells using a flow cytometer supporting five-color cytometry. Collect at least 50,000 total events for each sample. Analyze samples by flowjo or any software for flow cytometry data analysis. Gate viable cells and exclude cell debris. Plot this gate in PerCP x APC view to allow human cell chimerism to be visualized. Draw a gate to include cells that are human CD45 positive and mouse CD45 negative. Humanization levels are defined as the proportion of total nucleated cells that stain positive for human CD45 only. Plot the human CD45^+^ cells in a BV421 x PE view to visualize human T and B cell development. Draw a gate to include cells that are both CD3 and CD20 negative. Plot the CD3- CD20- cells in a FITC x PE view to visualize human natural killer cell (NK) cell and monocyte development.

### 3.3 Tumor engraftment in humanized NSG-SGM3 mice

All animal studies need to be performed in accordance with protocols approved by the Institutional Animal Care and Use Committee.

All procedures are performed under level 2 biosafety cabinet.

Transfect or infect ES A673 cells with luciferase expression vector (pMMP-Luc-Neo) or virus. The A673 cell line is often used to establish ES xenograft tumors in mouse models (15–17). Other cell lines such as TC71 and SK-ES that have previously proven engraftment (18, 19) can be utilized here as well. Select for luciferase positive cells using appropriate antibiotics (300 μg/mL neomycin). Resuspend selected cells in Matrigel at 1 x 10^7^ cells/mL. Keep on ice.

Use isoflurane to anesthetize humanized mice (>25% of human CD45^+^ cells in blood). Keep the animal anesthetized with isoflurane during the procedure. For orthotopic injection, lay the anesthetized animal on its side on the surgical bed, right tibia on the top. Use 70% Ethanol for aseptic preparation of tibia area. By a rotating action using a 26 Gauge needle, bore a hole through the notch in the knee into the tibia. Remove the needle carefully. Slowly and steadily inject the tumor cell suspension (1x10^5^ cells in 10 μL Matrigel) using a Hamilton syringe into the same hole. After injection, wipe the injection area with a Povidone-Iodine swab. For the subcutaneous injection, inject 1x10^6^ cells in 100 μL Matrigel, in both dorsal flanks of the mice. Place the animal back into the microisolator cage.

Monitor tumor growth by IVIS imaging once a week. Inject D-luciferin intraperitoneally into the transplanted mice. Image the mice according to the manufacturer’s instructions.

Validate tumor infiltration of human and mouse immune cells by immunofluorescent staining of freshly isolated tumor tissues. Embed the tissues in optimal cutting temperature compound and freeze at −80°C. Cut the frozen tissues into 10 μm sections using a cryostat microtome. Fix with 4% PFA or acetone, stain with appropriate antibodies (**Table 1**). Counterstain nuclei with DAPI in VECTASHIELD antifade mounting medium. Take fluorescent images using the EVOS M5000 imaging system or any fluorescent microscope with a camera.

### 3.4 Evaluation of immunotherapy (CD47 blockade) in humanized vs non-humanized ES xenograft NSG-SGM3 mice

Validate tumor engraftment on Day 4 after tumor cell injection using IVIS imaging.

Divide the tumor bearing mice randomly into two groups, IgG control group and magrolimab treatment group. For non-humanized mice with tumors, via intraperitoneal injection, administer 100 μg/animal of IgG or magrolimab every day on Days 4 to 10. For humanized mice bearing tumor, administer 6 μg/animal of IgG or magrolimab on Day 4, 12 μg/animal on Days 12-14, 25 μg/animal on Days 15-17, 50 μg/animal on Days 18-20 and 100 μg/animal on Days 21-22.

Monitor tumor growth by IVIS imaging once a week. Follow the animals until death or sacrificed upon reaching a tumor size of 2 cm in any dimension. Harvest the tumors and/or lungs for necessary subsequent analysis after sacrifice.

### 3.5 Statistics

For comparisons between two independent groups with approximately normally distributed variables, unpaired, parametric two group t-tests were used. Welch’s correction was used in case of unequal variances. Experiments with multiple independent groups were analyzed by analysis of variance (ANOVA). For studies where percentage of mice with lung metastasis was analyzed, Fisher’s Exact test was used. ANOVA was conducted using SAS 9.4 (SAS Institute), and Fisher’s Exact test was performed in GraphPad StatMate version 2.00. Sample size calculation was conducted using PASS 20 (Power Analysis and Sample Size Software. NCSS, LLC). All data are presented as the mean ± SD of at least three independent experiments except where stated.

## 4 Results

### 4.1 Establishment of humanized NSG-SGM3 mouse model utilizing CD34^+^ HSCs from fresh cord blood

**Figure 1:**
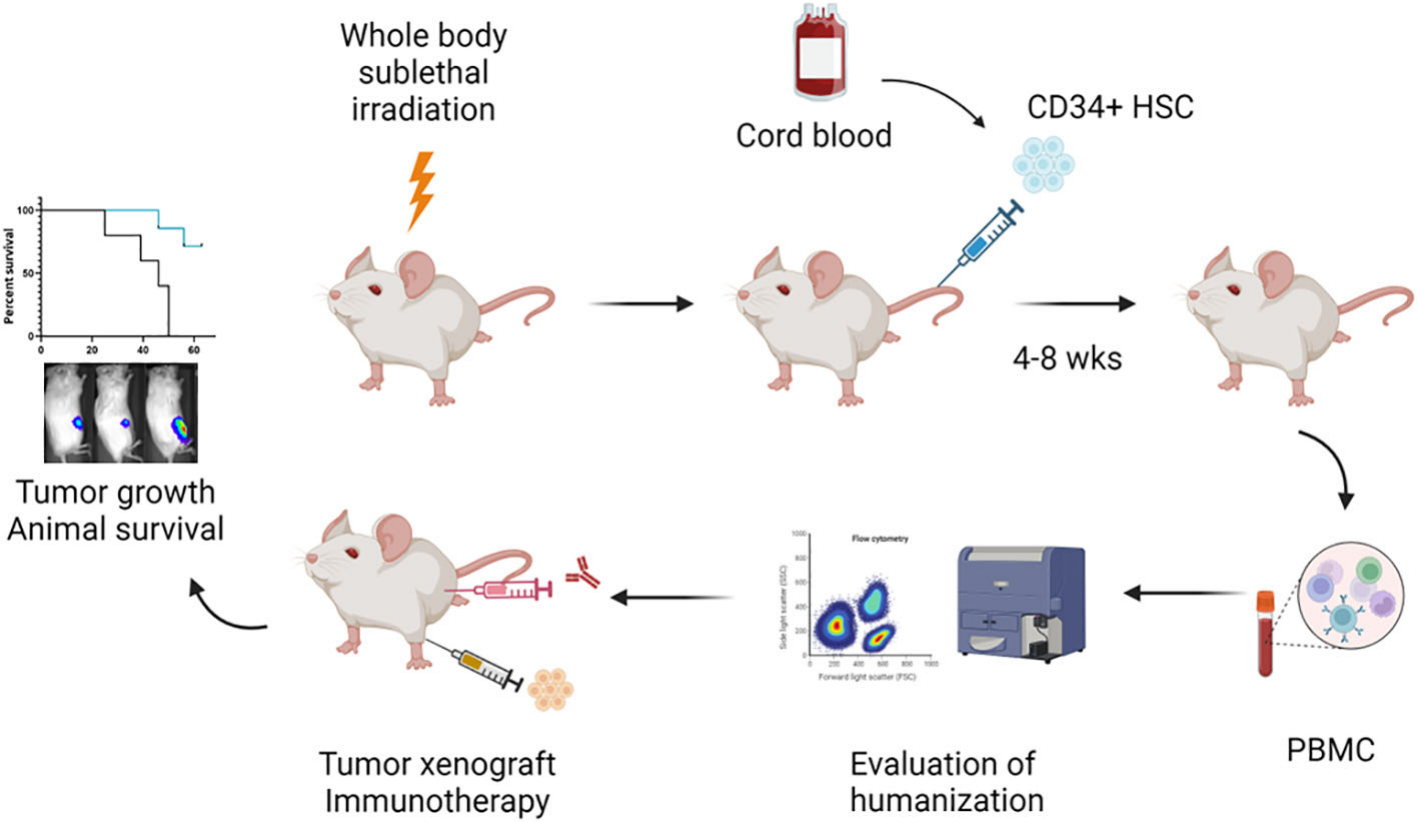
Schematic representation of the workflow to establish the orthotopic humanized mouse model for evaluation of immunotherapy in bone cancer. (Created by BioRender.com). In the current protocol, 3- to 4-week-old NSG-SGM3 mice are subjected to whole body sublethal irradiation 24 hours prior to the intravenous injection of fresh cord blood derived CD34+ HSCs. At 4 to 8 weeks post CD34+ HSC implantation, blood is drawn from the animals to evaluate the status of humanization by multi-color flow cytometry. Humanized mice (≥ 25% hCD45+ cells in the blood) are utilized for tumor cell engraftment and subsequent immunotherapy treatment. Tumor development including primary tumor growth and lung metastasis is monitored by IVIS imaging. Animal survival is followed until death or necessitating sacrifice.

**Figure 2:**
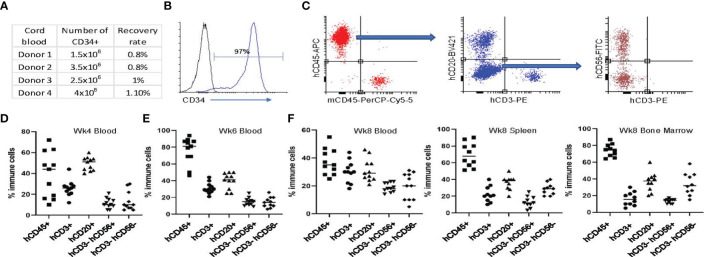
Establishing the humanized NSG-SGM3 mouse model utilizing CD34+ HSCs from fresh cord blood. **(A)** Summary of the number and recovery rate of CD34+ cells from healthy donors’ cord blood. **(B)** Representative flow cytometry analysis result showing the level of CD34 expression of CD34+ cells after fresh isolation from cord blood mononuclear cells. **(C)** Gating strategy for evaluating humanization efficiency and proportion of human immune cells in the CD34+ HSC transplanted animals. Plot the viable cells in PerCP x APC view to visualize human cell chimerism. Draw a gate to include cells that are human CD45 positive and mouse CD45 negative. Plot the human CD45+ cells in a BV421 x PE view to visualize human T and B cell development. Draw a gate to include cells that are both CD3 and CD20 negative. Plot the CD3- CD20- cells in a FITC x PE view to visualize human NK cell and monocyte development. **(D, E)** Evaluation of humanization status using peripheral blood at weeks 4 **(D)** and 6 **(E)** post hCD34+ HSC implantation. **(F)** Evaluation of humanization status using peripheral blood, spleen and bone marrow at week 8 post hCD34+ HSC implantation. Percentage of hCD45+ cells in total mononuclear cells and hCD3+, hCD20+, hCD3-hCD56+, hCD3-hCD56- cells in total hCD45+ cells are shown from 1 of 3 independent experiments with similar results.

A schematic of the workflow for the development of the humanized mouse model for bone cancer (Ewing sarcoma) is shown in **Figure 1**. We isolated hCD34^+^ HSCs from fresh cord blood units and found that the recovery rate of CD34^+^ HSCs was around 1% of the number of mononuclear cells (**Figure 2A**). The purity of the CD34^+^ HSCs was > 95% (**Figure 2B**). Human immune cell reconstitution was characterized by multicolor flow cytometry of the blood, spleen and bone marrow at different time points with the gating strategy as shown in **Figure 2C**. We found that at week 4 post engraftment, 10-70% of total leukocytes in the recipient mice were human CD45^+^ (hCD45^+^) cells. In these hCD45^+^ cells, around 25% were hCD3^+^ T cells and 50% were hCD20^+^ B cells. In addition, approximately 10% of the hCD45^+^ cells were hCD3^-^ hCD56^+^ NK cells and hCD3^-^ hCD56^-^ monocytes (**Figure 2D**). At week 6, the average percentage of hCD45^+^ cells had increased to 80%. In these human leukocytes, approximately 30%, 40%, 15% and 10% were hCD3^+^ T, hCD20^+^ B, hCD3^-^ hCD56^+^ NK and hCD3^-^ hCD56^-^ monocytes, respectively (**Figure 2E**). When we evaluated the status of humanization at week 8, the percentage of hCD45^+^ cells were between 25-55% in the blood and an average of 70% in the spleen and bone marrow. The percentage of T, B, NK and monocytes were around 20-30% in the blood and 15-40% in the spleen and bone marrow (**Figure 2F**). Taken together, the engraftment of CD34^+^ HSCs from fresh cord blood successfully induced robust and early reconstitution of human immune system with major subpopulations of immune cells in the NSG-SGM3 host.

### 4.2 Establishment of ES xenograft tumors in humanized NSG-SGM3 mice

**Figure 3:**
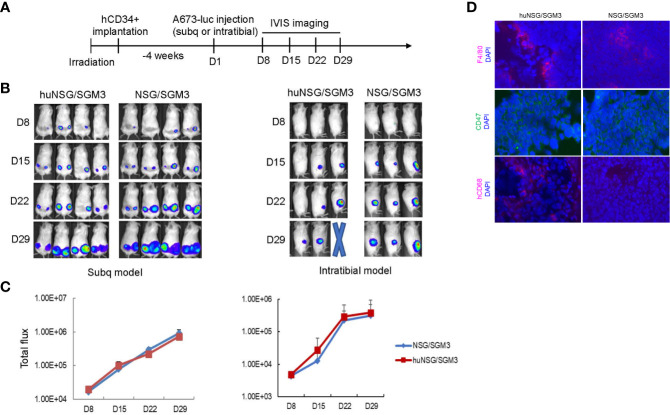
Establishing an orthotopic ES xenograft mouse model in humanized NSG-SGM3 mice. **(A)** Experimental timeline showing that at 4 weeks post hCD34+ HSCs implantation, the humanized mice were injected with luciferase expressing ES A673 cells subcutaneously into the flanks or orthotopically into the tibia. Tumor development was monitored by IVIS imaging once a week. **(B)** Representative IVIS images of mice showing comparison of the tumor growth in humanized (huNSG-SGM3) and non-humanized (NSG-SGM3) mice in both the subcutaneous (left) and the intratibial (right) injection models. **(C)** Comparison of growth curves of tumors in huNSG-SGM3 and NSG-SGM3 mice in both subcutaneous (left) and intratibial (right) models. **(D)** Immunofluorescent staining of tumor tissues from huNSG-SGM3 and NSG-SGM3 mice. F4/80+ mouse macrophages and CD47+ tumor cells were shown in both huNSG-SGM3 and NSG-SGM3 tumor tissues. hCD68+ human macrophages were only shown in tumors from huNSG-SGM3 mice.

To investigate whether human immune cell reconstitution results in any difference in the engraftment rate of tumors, we injected patient derived ES cells subcutaneously into the flanks or orthotopically into the tibia of both humanized (>25% hCD45^+^ leukocytes in total leukocytes) (huNSG-SGM3) and non-humanized (no CD34^+^ HSCs engrafted) NSG-SGM3 mice. We performed the injections at 4 weeks post hCD34^+^ HSC implantation (**Figure 3A**) because the majority of the mice had more than 25% hCD45^+^ leukocytes at that time point. Moreover, ES is predominantly a cancer that occurs in children and adolescents, engraftment of the tumors in young mice will more closely resemble the age of disease onset in humans. We found the tumor engraftment rates were similar comparing huNSG-SGM3 and their non-humanized counterparts by either route of administration (subcutaneous or intratibial) (**Figure 3B**). No significant difference in tumor growth between humanized and non-humanized mice was noted at time points of week 1-4 post tumor cell implantation (**Figure 3C**). We compared the infiltration of immune cells in the xenograft tumors harvested from the huNSG-SGM3 and non-humanized mice. While murine macrophages (F4/80^+^) were present in both humanized and non-humanized mice tumors, we detected human macrophages (hCD68^+^) (**Figure 3D**) in tumor tissues from huNSG-SGM3 but not non-humanized mice. No T or NK cells were identified in either tumor tissues. These results are consistent with the previous findings that tumor associated macrophages are predominant while few cytotoxic T or NK cells are found in ES patient tumors (20).

### 4.3 Evaluation of CD47 blockade in the humanized NSG-SGM3 ES orthotopic mouse model

**Figure 4:**
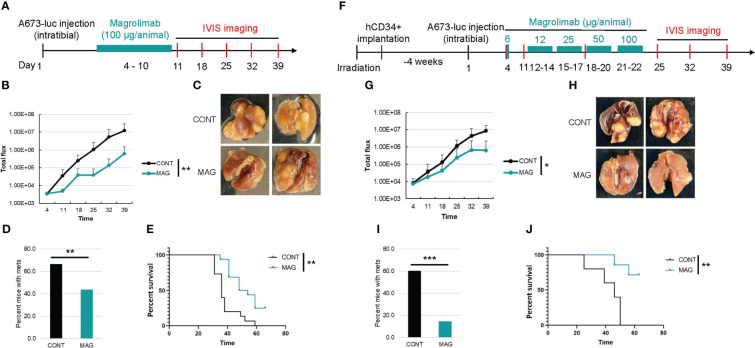
Comparison of the efficacy of CD47 blockade in non-humanized and humanized orthotopic mouse model of ES. **(A)** Experimental timeline showing that the non-humanized mice were injected with luciferase expressing A673 cells orthotopically into the tibia and the tumor bearing mice were treated with control IgG or magrolimab (MAG) (100 ug/animal, i.p.) from day 4-10 post A673 injection. Tumor development was monitored by IVIS imaging once a week. **(B)** Primary tumor growth curves in non-humanized NSG-SGM3 mice. **(C)** Representative images of lungs from the mice in control or MAG treated groups. **(D)** Percentage of mice with lung metastasis in control or MAG treated groups in non-humanized NSG-SGM3 mice. **(E)** Kaplan-Meier curves showing the survival of the control or MAG treated non-humanized NSG-SGM3 mice. **(F)** Experimental timeline showing that 4 weeks after hCD34+ HSCs implantation, the humanized mice were injected with luciferase expressing A673 cells orthotopically and the tumor bearing mice were treated with control IgG or MAG at a priming dose of 6 ug/animal followed by dose escalation from 12-100 ug/animal from day 12-22 post A673 injection. Tumor development was monitored by weekly IVIS imaging. **(G)** Growth curves of primary tumors in control or MAG treated huNSG-SGM3 mice. **(H)** Representative images of lungs from the huNSG-SGM3 mice in control or MAG treated groups. **(I)** Percentage of mice with lung metastasis in control or MAG treated groups in huNSG-SGM3 mice. **(J)**, Kaplan-Meier survival curves of the control or MAG treated huNSG-SGM3 mice. *P<0.05, **P<0.01, ***P<0.001.

To validate the humanized NSG-SGM3 mouse model that we established in evaluating immunotherapy, we compared the efficacy of a potentially effective immunotherapy in treating ES xenograft tumors in humanized and non-humanized NSG-SGM3 mice. It was reported that neither checkpoint blockade nor T cell therapy is effective in ES in large part due to the low mutational burden and MHC expression level in ES (21, 22). Macrophages are the most abundant immune cells in ES tumor microenvironment and confer a poor prognosis (23) by potentially contributing to ES cell survival and dissemination. We found that CD47, a “don’t eat me” signal that tumor cells upregulate to escape macrophage mediated phagocytosis, is highly expressed on ES (**Figure 3D**). We hypothesize that blocking the CD47 signal may overcome macrophage mediated immune evasion in ES and therefore constitute an effective immunotherapy approach. To this end, we utilized a humanized IgG4 antibody that targets CD47, magrolimab, which is currently in phase III clinical trials for treating AML/MDS but has not yet been investigated in ES. According to the schedule shown in **Figure 4A**, we administered magrolimab at the dose level of 100 μg/animal once daily for a week after intratibial establishment of the ES xenograft tumors in NSG-SGM3 mice. We found the mice tolerated well with this dose, and magrolimab treatment significantly slowed down tumor growth compared to the control IgG treated condition (**Figure 4B**). Furthermore, magrolimab significantly reduced lung metastasis (**Figure 4C, D**) and prolonged NSG-SGM3 mice survival (**Figure 4E**). Interestingly, when we administered the same dose (100 μg/animal) to the tumor-bearing humanized NSG-SGM3 mice, the animals died after a single injection. Complete blood count (CBC) analysis revealed that these mice are anemic (**Table 3**). Recent clinical safety evaluation data showed that magrolimab was well tolerated using a priming dose at 1 mg/kg followed by maintenance doses ranging from 3 to 45 mg/kg (24). Using a dose de-escalation experiment, we determined that 6 μg/animal is the priming dose of magrolimab in the humanized NSG-SGM3 mice. We then followed a dose escalation schedule from 12 to 100 μg/animal for the rest of the treatment (**Figure 4F**). We found that magrolimab treatment also significantly reduced tumor growth in humanized NSG-SGM3 mice (**Figure 4G**). Importantly, magrolimab was more effective in significantly limiting lung metastasis (**Figures 4H, I**) and extending animal survival (**Figure 4J**) in humanized NSG-SGM3 compared to the non-humanized mice (**Figures 4C-E**).

**Table 3:** Complete blood count from selected humanized NSG-SGM3 mice.

	Mouse 1	Mouse 2	Mouse 3	Mouse 4
RBC (106/mm3)	7.27	6.95	0.83	1.23
HGB (g/dl)	12.8	13	1.6	2.2
HCT (%)	37.8	38.6	4.6	6.5
MCH (pg)	17.6	18.7	0	18
MCHC (g/dl)	33.9	33.6	0	33.7
PLT (103/mm3)	467	518	119	106

RBC, red blood cells; HGB, hemoglobin; HCT, hematocrit; MCH, mean corpuscular hemoglobin; MCHC, mean corpuscular hemoglobin concentration; PLT, platelets.

## 5 Discussion

In this protocol, we describe the generation of a humanized orthotopic mouse model of ES in young NSG-SGM3 mice using intravenous injection of cord blood derived CD34^+^ HSCs followed by intratibial transplantation of ES patient derived cells (**Figure 1**). We showed that this model is suitable for pediatric cancer research because human immune cells were reconstituted early in young mice (**Figure 2**). Furthermore, utilizing orthotopic transplantation of tumor cells, this model enables mimicking of bone cancer tumorigenesis and metastasis in the appropriate microenvironment in the bone (**Figures 3**, **4**). Importantly, we believe this model is an improved model compared to the non-humanized model in evaluating the safety and efficacy of immunotherapy regimens as evidenced by the results of our investigation with CD47 blockade (**Figure 4**). With careful design and optimization, this model can be utilized for other pediatric and/or bone cancers.

In optimization of humanized models, the particular experimental objective and the hypothesis to be tested need to be taken into account. To optimize the model for pediatric cancer which requires early reconstitution of human immune system in young mice, we considered several variables including choice of the host, age of the recipient mice, method of human immune cell engraftment and dose of the human cells. We found that both NSG and NSG-SGM3 enable reconstitution of human immune cells but NSG-SGM3 specifically allows relatively more robust reconstitution of myeloid lineage cells as previously reported (25, 26). Because myeloid cells including macrophages are predominant immune cell population in ES tumor microenvironment (20), we chose NSG-SGM3 as the host. In addition to the NSG strains, NOG (NOD.Cg-PrkdcscidIL2rgtm1Sug), BRG (Balb/c Rag2-/-IL2rg-/-) and recently developed NPG (NOD-PrkdcscidIL2rgnull) and NCG (NOD-Prkdcem26IL2rgem26Nju) are popular strains most frequently used in the models for recapitulation of human immune system. Among these strains, NOG-EXL and MISTRG are also optimal for human myeloid cell reconstitution. Several reviews have given excellent overviews of immunodeficient hosts for humanized mouse models for cancer immunotherapy (27, 28).

When determining whether to use newborn, juvenile or adult mice for human HSC engraftment, in addition to cancer type, practical considerations need to be taken into account. To study pediatric cancer, we utilized 3-week-old mice which is the youngest mice commercially available. Using newborn engraftment protocol is another option in which humanized immune system can be developed at a younger age. However, breeder cages need to be set up in-house and closely monitored for multiple new litters within a narrow time frame for enough pups needed for experiments. Another drawback for the newborn approach is that the timing of newborn litter needs to be coordinated with the availability of the source of human HSCs such as cord blood which represents a challenge sometimes.

Humanized mice can be generated using various approaches among which the two most common ones include a) engrafting human PBMC isolated from peripheral blood into low dose irradiated or non-irradiated immunodeficient mice by intraperitoneal (IP), intravenous (IV) or intrasplenic (IS) administration; b) engrafting human HSCs isolated from either adult peripheral blood, bone marrow or cord blood, or from fetal liver into low dose irradiated immunodeficient mice by IP or IV administration (29). In this protocol, we chose the HSCs engraftment method because its advantage in superior engraftment efficiency and reconstitution potential and low induction of a xenogeneic graft versus host disease compared to the PBMC approach. We compared the engraftment capability of freshly isolated or frozen-thawed CD34^+^ HSCs from fresh cord blood and mobilized peripheral blood. We found that only the engraftment of freshly isolated cord blood CD34^+^ HSCs at dose of ≥ 2 x 10^5^ cells/animal enables the robust reconstitution of human immune system (> 50% hCD45^+^ cells in the blood) in young NSG-SGM3 mice (4 weeks after engraftment). Although we observed variability of engraftment rate depending on the source and donor of cord blood, we alleviated the effects of this variation on experimental results by dividing the humanized mice derived from different cord blood donors evenly into each treatment group in all experiments.

As with any animal models, this model has advantages and limitations in its recapitulation of the human patient tumor microenvironment. The advantages include early onset of reconstitution of human immune system for pediatric cancer research and efficient reconstitution of human innate immune system including NK cells and myeloid lineage cells for evaluation of innate immune cell associated immunotherapy. In addition, this model showed correlation of the tested immunotherapy regimen to its performance in the clinic. We showed evidence that the CD47 blockade alone had statistically significant but limited efficacy in controlling ES development (**Figure 4**) which is consistent with previous clinical trial results that CD47 blockade alone is not sufficient to control disease development in solid tumors (30). Importantly, our model showed the same indication of the dose dependent toxicity associated with the CD47 blockade as in clinical trials in patients with acute myeloid leukemia or myelodysplastic syndrome (31). This toxicity was specific to CD47 blockade and was not induced by the control IgG antibody or other treatments such as doxorubicin (data not shown). It was reported that NSG-SGM3 mice develop anemia upon humanization with cord blood CD34^+^ HSCs which shortens the lifespan of humanized NSG-SGM3 mice and poses a challenge for immuno-oncology research that requires a long study window (30, 32). However, for an aggressive cancer such as ES, this is not an issue. We observed the same anemic phenotype in these mice, but they remain alive and well for at least 16 weeks after human HSCs engraftment. The primary ES xenograft tumors quickly grow to the size that necessitates sacrifice or metastasize to the lung that causes death of the animal 8 weeks after tumor implantation if not sooner. If a long study window is required, the NSG-SGM3 mouse host can be replaced by NSG or other mouse strains. An inherent limitation of the current model is that patient derived xenograft tumors cannot be directly transplanted orthotopically due to the limited space in the site of administration (tibia). However, patient derived xenograft tumor tissues can be digested, and tumor cells isolated, before injecting into the animal orthotopically. Finally, like other humanized mouse models, this model is limited by the relatively high cost and technical challenges compared to syngeneic or transgenic mouse models. However, compared to clinical trials in human patients, our humanized model is only a fraction of the cost of a clinical trial. Furthermore, our humanized mouse model offers sufficient sample size to ensure statistical power for analysis while patient recruitment in clinical trials in rare cancers such as ES is often a challenge.

It is important to note that humanized mouse models are not perfect “avatar” for humans. Efforts are needed to continue improving and tailoring these models for studying individual types of human cancer or testing specific hypothesis. A potential improvement or modification to the current model is utilizing immunodeficient mice harboring a mutation in the c-kit gene (encodes SCF receptor or CD117) (NSGW41 or NBSGW) as an alternative host to support human myeloid reconstitution without pre-conditioning (33–36). These mice do not require irradiation prior to engraftment and have increased human erythropoiesis and HSC longevity (32, 37, 38). Another approach to improving the condition of anemia in this model is expressing cytokines erythropoietin and IL-3 via knock-in or hydrodynamic injection of DNA plasmids as previously reported (39) for increased reconstitution and development of human erythrocytes. The current model is tailored to be best suited for cancers whose tumor microenvironment is dominated by myeloid lineage cells. For cancer types in which T or B cells play more critical roles, the model needs to be modified to support more robust reconstitution of these immune cells. For example, humanized mouse models made by engrafting human fetal or neonatal thymus and HSCs into immunodeficient mice have robust reconstitution of functional human T cells that have matured in the presence of human self-peptides and human leukocyte antigen molecules (40, 41). However, these models may be limited by the availability of fetal or neonatal tissues. All in all, with individual cancer type and specific tumor microenvironment taken into account, the current humanized orthotopic mouse model can be modified for other pediatric and/or bone cancers.

## Data availability statement

The raw data supporting the conclusions of this article will be made available by the authors, without undue reservation.

## Ethics statement

The animal study was approved by The New York Medical College Institutional Animal Care and Use Committee. The study was conducted in accordance with the local legislation and institutional requirements.

## Author contributions

WL: Conceptualization, Validation, Writing – review & editing, Data curation, Formal Analysis, Methodology, Writing – original draft. HH: Data curation, Formal Analysis, Methodology, Writing – review & editing. YL: Data curation, Formal Analysis, Methodology, Writing – review & editing. JP: Data curation, Formal Analysis, Methodology, Writing – review & editing. JA: Supervision, Writing – review & editing. MC: Conceptualization, Funding acquisition, Project administration, Supervision, Validation, Writing – review & editing.
